# Electro-acupuncture for long COVID neuropsychiatric symptoms: study protocol for a prospective, randomized sham-controlled, patient-assessor-blinded clinical trial

**DOI:** 10.3389/fmed.2025.1620288

**Published:** 2025-09-04

**Authors:** Dong-jue Wei, Chun-wai Chow, William Y. H. Cheung, Wing-fai Yeung, Pei-hua Cao, Ching Liong, Hai-yong Chen, Shipping Zhang, Linda L. D. Zhong

**Affiliations:** ^1^School of Chinese Medicine, Hong Kong Baptist University, Hong Kong, China; ^2^School of Nursing, The Hong Kong Polytechnic University, Hong Kong, China; ^3^Clinical Research Center, Zhujiang Hospital, Southern Medical University, Guangzhou, China; ^4^Chung Chi College, The Chinese University of Hong Kong, Hong Kong, China; ^5^School of Chinese Medicine, Li Ka Shing Faculty of Medicine, The University of Hong Kong, Hong Kong, China; ^6^School of Biological Sciences, Nanyang Technological University, Singapore, Singapore

**Keywords:** long COVID, electroacupuncture, neuropsychiatric symptoms, randomized clinical trial, protocol

## Abstract

**Introduction:**

Patients recovering from long COVID often endure a spectrum of neuropsychiatric symptoms, including cognitive impairment, memory deficits, mood disturbances and sleep disorders, that significantly impact their quality of life. Acupuncture, particularly electroacupuncture, has shown promise in addressing these symptoms. Currently there is no high-quality clinical trial for acupuncture on long COVID neuropsychiatric symptoms.

**Methods and analysis:**

In this 24 weeks, sham-controlled, patient-assessor-blinded randomized trial, 150 long COVID patients will be equally allocated to either an electroacupuncture group (EAG) or a sham control group (SCG). Each subject will receive a total of 32 intervention sessions over a 16 weeks intervention phase (two sessions each week) and will be followed up for an additional 8 weeks. Primary outcomes include changes in the Mini-Mental State Examination (MMSE) and the Chinese version of the Beck Depression Inventory (CBDI) scores. Secondary outcomes include the Insomnia Severity Index (ISI), Brief Fatigue Inventory-Taiwan (BFI-T), and the Short Form 12 (SF-12). All outcomes will be assessed at baseline and then at 4 weeks intervals during both the treatment and post-treatment periods.

**Discussion:**

This trial aims to generate robust clinical data on the therapeutic effects of electroacupuncture for long COVID. The anticipated results will clarify electroacupuncture’s value as a therapeutic option for neuropsychiatric symptoms in long COVID patients, contributing to evidence-based practice in integrative medicine.

## Introduction

Since SARS-CoV-2 first emerged in late 2019, COVID-19 has profoundly disrupted both public health and socioeconomic systems around the globe. Although the World Health Organization (WHO) officially ended the global public health emergency on 5 May 2023, chronic health issues stemming from COVID-19 continue to affect many recovering patients. These lingering symptoms, which emerge 3 months after infection, persist for a minimum of 2 months, and cannot be attributed to alternative diagnoses, are classified by the WHO as post-COVID-19 condition, or long COVID (1). Epidemiological studies from various countries report high prevalence rates of long COVID (2–9), marking it as a pressing public health issue. This condition involves a broad spectrum of multisystem complaints, predominantly severe fatigue, exertion-related malaise, shortness of breath, palpitations, brain fog, and sensory disturbances such as loss of taste or smell (10–14).

Among the various symptoms triggered by long COVID, neuropsychiatric symptoms have garnered significant attention, including cognitive impairment (15–18), persistent fatigue, anxiety, and depression (10–14). The potential mechanisms underlying long COVID remain unclear, with prevailing theories including persistent viral reservoirs (19), persistent inflammation (20), sustained autoimmune responses (21), host microbiome factors (22, 23), and endothelial dysfunction leading to subsequent blood coagulation (24). However, these theories are still in the hypothesis stage, and effective treatments remain lacking. While existing approaches such as cognitive behavioral therapy (CBT) and nasal irrigation provide some symptom relief (25–27), their efficacy is inconsistent and often associated with side effects, highlighting the urgent need to develop safe and effective non-pharmacological therapies to address the potential long-term impacts of long COVID.

Acupuncture, a widely used technique in traditional Chinese medicine (TCM), is commonly applied in the treatment of neurological and neuropsychiatric disorders. Previous studies have shown that acupuncture can regulate central nervous system activity, thereby improving emotional issues such as anxiety and depression (28, 29). Additionally, acupuncture has demonstrated significant efficacy in treating fatigue syndrome and insomnia (30–32). During the pandemic, acupuncture was employed as an adjunctive treatment in China with formal clinical guidelines (33). Collectively, these results imply that acupuncture could serve as a potential therapeutic approach in managing long COVID. Within acupuncture stimulation protocols, both manual needling and electrical stimulation at acupoints are routinely employed (34). And electroacupuncture has shown unique advantages in enhancing neural regulation and mitigating cognitive impairments associated with central nervous system disorders (35, 36). Pei (37) demonstrated that electroacupuncture substantially alleviated deficits in spatial memory. Electroacupuncture can significantly making its intervention in neuropsychiatric symptoms more targeted (38). However, there is currently no electroacupuncture research addressing neuropsychiatric symptoms in long COVID, and high-quality clinical evidence is lacking.

Building on this, the present study employs a prospective, sham-controlled clinical protocol with participants randomly allocated to receive electroacupuncture within a TCM-guided individualized treatment framework, to rigorously assess its therapeutic impact on neuropsychiatric manifestations of long COVID. The innovation of this study lies in its integration of individualized treatment concepts with randomized controlled trial design, aiming to provide a safe and effective non-pharmacological intervention option for long COVID patients and to advance the clinical application of acupuncture in managing neuropsychiatric symptoms in the post-pandemic era.

## Methods and analysis

### Trial design and setting

**FIGURE 1:**
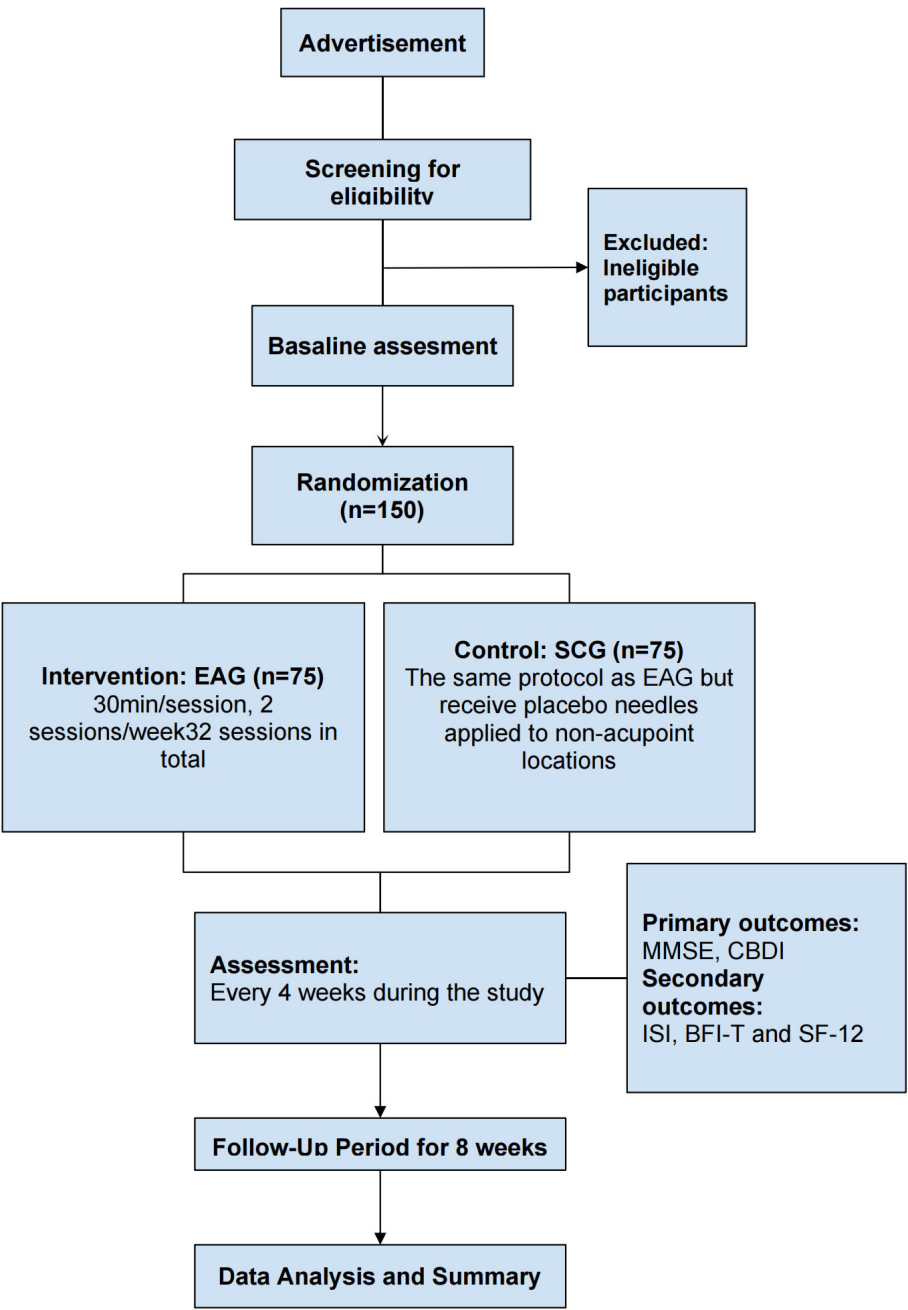
Trial design flow chart.

This is a prospective, patient-assessor-blinded, randomized, sham-controlled, multi-center trial designed to examine electroacupuncture’s impact on neuropsychiatric sequelae in long COVID survivors. A total of 150 eligible participants will be allocated in equal numbers to either the electroacupuncture group (EAG) or the sham control group (SCG). Each cohort will complete 32 intervention sessions over a 16 weeks period (2 sessions weekly) and will be followed up for an additional 8 weeks. The primary outcomes will be the changes in the Mini-Mental State Examination (MMSE) and the Chinese Beck Depression Inventory (CBDI) scores before and after treatment. Secondary outcomes will include changes in the Insomnia Severity Index (ISI), Brief Fatigue Inventory-Taiwanese (BFI-T), and the 12-item Short-Form Health Survey (SF-12) scores during the treatment and follow-up periods. These questionnaires (Appendix 1) will be assessed every 4 weeks during both the treatment and follow-up periods. The schedule detailing participant enrolment, intervention administration and outcome assessments are summarized in Table 1, while a flow diagram of the trial design is presented in Figure 1. This trial has been registered in clinicaltrials.gov (NCT05890508) and approved by the Research Ethics Committee of Hong Kong Baptist University (REC/21-22/0467).

**TABLE 1:** Schedule for outcome measurement.

Period	B	T																F	
**Visit**	**1**				**2**				**3**				**4**				**5**	**6**	**7**
**Week**	**0**	**1**	**2**	**3**	**4**	**5**	**6**	**7**	**8**	**9**	**1** **0**	**1** **1**	**1** **2**	**1** **3**	**1** **4**	**1** **5**	**1** **6**	**2** **0**	**2** **4**
General assessment	√																		
Screening for enrolment	√																		
Treatment (acupuncture/sham-acupuncture)	Twice a week (16 week treatment)																		
MMSE	√				√				√				√				√	√	√
CBDI	√				√				√				√				√	√	√
ISI	√				√				√				√				√	√	√
SF12	√				√				√				√				√	√	√
Adverse event report	As needed																		

B, baseline; T, treatment phase; F, follow-up phase; MMSE, Mini-Mental State Examination; CBDI, Chinese Beck Depression Inventory Fast Screen; ISI, Insomnia Severity Index; SF12, the Short Form 12.

### Sample size

The target sample size was determined by reviewing prior trials using MMSE and BDI to assess the effectiveness of acupuncture versus sham treatment. The observed differences in MMSE and BDI scores between groups were 2.82 and 3.96 points, respectively (39, 40). Based on effect sizes of 0.76 for MMSE and 0.75 for BDI, the BDI-based between-group difference was smaller and was therefore selected for estimation of sample size to yield a more conservative result. To control the overall type I error and ensure sufficient statistical power, the following parameters were applied: a two-sided α level of 0.025 (Bonferroni correction), a power of 0.8, and an equal allocation ratio (1:1) between the acupuncture and sham acupuncture groups. Under these assumptions, the calculation yielded a requirement of 36 subjects per arm. Allowing for an anticipated 20% dropout rate, the target total sample size was adjusted to 90 cases. To increase robustness and ensure an adequate sample reserve for future subgroup analyses, we plan to recruit 150 participants (41–44). Sample size estimation was conducted using G*Power (Version 3.1).

### Randomization and blinding

Participants will be randomized in a 1:1 ratio to either the EAG or SCG. Those allocated to the active treatment arm will undergo standardized electroacupuncture sessions, while the control arm will receive an equivalent sham procedure. A computer-generated simple randomization sequence will be created prior to the study, without stratification or block assignment. The randomization procedure will be conducted by the research unit of the School of Chinese Medicine, Hong Kong Baptist University. Allocation codes will be placed in sequentially numbered, opaque, tamper-evident envelopes and stored in a locked cabinet, with sole key custody maintained by the Principal Investigator (PI). All participants, outcome assessors, statisticians, and all research staff involved in patient contact-except the acupuncturists delivering the treatments, will remain blinded to treatment assignment. To assess the success of blinding, the James Blinding Index will be used at the end of the study. Emergency unblinding is allowed if serious adverse events arise and immediate measures are required to ensure participant safety. Any request for emergency unblinding must be submitted to the PI by co-investigator. Appropriate medical interventions will be provided promptly, and the reasons for unblinding will be documented in detail, including the date and signature of the responsible investigator. The participant involved will be withdrawn from the study.

### Participants

Participants will be enrolled through two primary channels: (1) newspaper advertisements inviting interested individuals to contact the research team via telephone; or (2) referrals from clinics affiliated with the Hong Kong Hospital Authority. The relevant clinical teams will first contact potential participants, after which the research staff will conduct eligibility screenings. During the initial consultation, a physician (co-investigator) will conduct a detailed clinical history review and comprehensive examination. the PI or a designated co-investigator will furnish potential participants with detailed information regarding the study objectives, methodology, and any risks associated with acupuncture. Written informed consent (Appendix 2: Participant Consent Form) will be obtained prior to participation. Participants will also be informed of their right to withdraw at any point during the study. A total of 150 long COVID patients with neuropsychiatric symptoms will be recruited. The study will be conducted across six Chinese Medicine Research and Clinical Centers of Hong Kong Baptist University. Eligibility criteria are detailed in Table 2.

**TABLE 2:** Inclusion and exclusion criteria.

Inclusion criteria	Exclusion criteria
• Aged 18–80 years.**** • History of SARS-CoV-2 PCR + at least 4 weeks prior; PCR-negative at study entry.**** • At least one persistent neurological symptom (e.g., impaired concentration, headache, sensory disturbances, depression, or “brain fog”) starting around acute COVID-19.**** • Able and willing to consent and complete all assessments and procedures.	• Chronic/remote neurological disorders (e.g., stroke, head trauma, epilepsy, tumor). • Pre-existing intellectual disability. • Cardiovascular diseases (e.g., arrhythmia, heart failure, myocardial infarction, pacemakers). • Acute brain injury or encephalopathy from non-COVID causes (e.g., sepsis, organ failure, drug toxicity). • Documented pre-existing psychiatric illness, including substance abuse. • Open-heart surgery or cardiac arrest within the last 6 months. • Current hospitalization. • Pregnancy.
**Withdrawal criteria**	
• Confirmation of pregnancy during the trial.**** • Discovery of a disease that may affect the trial.**** • Problems with the medical or trial procedures.**** • Other reasons determined by the research team.	

### Participant retention and follow-up

This is a 24 weeks clinical trial, which subjects will need to take 32 sessions of acupuncture treatment and seven regular visits (treatment and follow-up). To bolster participant adherence, we will employ a three-pronged strategy. First, during the informed-consent interview, the research team will review the study timeline, outline possible adverse effects, clarify participant obligations, and provide continuous support and reassurance. Second, a two-week run-in period will screen out ineligible or poorly compliant volunteers before randomization. Third, a dedicated email address (cmcs@hkbu.edu.hk) and a direct telephone hotline will be activated to ensure prompt, proactive communication and address any participant queries throughout the trial.

### Handling of withdraw and dropout

Participants who meet withdrawal criteria or voluntarily withdraw from the study will have their reasons documented whenever possible. Data collected before withdrawal will be retained for analysis under the intention-to-treat principle unless consent is specifically withdrawn for data usage. Efforts will be made to minimize dropout through proactive follow-ups, flexible scheduling, and continuous communication via the designated email and hotline. Adverse events (AEs) or concerns will be addressed promptly, and any serious issues will be reported to the PI and the ethics committee. Withdrawals and dropouts will be transparently reported in the final study results to ensure data integrity and scientific rigor.

### Interventions

Following baseline assessment, participants randomized to the EAG will receive electroacupuncture stimulation from a registered Chinese Medicine practitioner with more than 5 years of Chinese medicine university education and more than 5 years of clinical experience. A systematic review (45) identified GV20 (Baihui), EX-HN1 (Sishencong), EX-HN3 (Yintang), SP6 (Sanyinjiao), ST36 (Zusanli), ST40 (Fenglong), and LR3 (Taichong) as the most frequently used acupuncture points for cognitive impairment. Accordingly, the rationale for choosing the acupoints is as follows: the acupoints on the head such as GV20 (Baihui) and EX-HN1 (Sishencong) have the local effectof regulating the brain function; the other acupoints on the lower limb, such as LR3 (Taichong) and ST36 (Zusanli), have the effect of soothing liver and strengthening spleenis (46). All acupoint locations adhere to WHO standard definitions (47), specific details of acupoints are summarized in Table 3 and Appendix 3.

**TABLE 3:** Locations of acupuncture points used in electroacupuncture group (EAG).

**Acupoint**	**Location**
Baihui (GV20)	On the top of the head, 7 B-cun directly superior to the posterior hairline, on the anterior median line.
Sishencong (EX-HN1)	Around Baihui (GV20), 1 B-cun anterior, posterior, and lateral to it.
Yintang (EX-HN3)	On the forehead, at the midpoint between the medial ends of the two eyebrows.
Sanyinjiao (SP6)	On the tibial aspect of the leg, posterior to the medial border of the tibia, 3 B-cun superior to the prominence of the medial malleolus.
Zusanli (ST36)	On the anterolateral aspect of the leg, 3 B-cun inferior to Dubi (ST35) and 1 fingerbreadth lateral to the anterior crest of the tibia.
Fenglong (ST40)	On the anterolateral aspect of the leg, 8 B-cun superior to the prominence of the lateral malleolus and two fingerbreadths lateral to the anterior crest of the tibia.
Taichong (LR3)	On the dorsum of the foot, between the first and second metatarsal bones, in the depression distal to the junction of the bases of the two bones, over the dorsalis pedis artery.

- EAG: electroacupuncture will be performed using single-use, sterile acupuncture needles with a diameter of 0.20 mm. Needles will be inserted to a depth of 1–3 cm, based on the thickness of local tissue, in accordance with traditional TCM standards. The acupoints are subjected to 2–5 Hz electroacupuncture from an electroacupuncture device (Hwato, Electronic Acupuncture Treatment Instrument, Suzhou Medical Appliance Factory, Jiangsu, China) at an intensity (5–10 mA) that can produce a muscle twitch acceptable to the participant. Each participant allocated to the EAG will undergo 30 min electroacupuncture sessions twice weekly throughout the four-month intervention period (48).
- SCG: for the sham-control arm, Streitberger’s non-invasive acupuncture needles (Gauge 8 × 1.2/0.30 × 30 mm) will be positioned at non-acupoint sites approximately 0.5 cun from the corresponding acupoints, replicating the same insertion procedure as the acupuncture group (49). Additionally, the needles will be connected to inactive output ports on the electroacupuncture device. The stimulator will emit only the same beeping sound and flashing light continuously, thus producing a “pseudo stimulation” setup that maintains blinding integrity without delivering a therapeutic effect. This sham procedure has been previously validated for both its credibility and its ability to maintain participant blinding (50–54).

### Outcomes measurement

#### Primary outcome

- Mini-Mental State Examination (MMSE)

Cognitive function will be evaluated using the Chinese version of MMSE scale (45), which assesses five domains: orientation (up to 10 points), memory (6 points), attention and calculation (5 points), language (8 points), and visuoconstructional ability (1 point). Total scores range from 0 to 30, with 21–24 indicating mild impairment, 10–20 moderate, and below 10 severe. Assessments will be performed at baseline, at weeks 4, 8, 12, and 16 (end of the intervention), and again 2 months after completion of treatment.

- Chinese Beck Depression Inventory (CBDI)

Depressive symptoms will be measured using the CBDI, a validated self-reported questionnaire comprising 21 items (maximum total score = 63) (55), with score ranges of 14–19 indicating mild, 20–28 moderate, and above 29 severe depression. Assessments will occur at baseline, at week 4, 8, 12, 16 (end of treatment) and again two months post-intervention.

#### Secondary outcome

Secondary endpoints include the score of ISI (56), BFI-T Form (57), and SF12 (58). All scales are assessed at baseline, at four-week intervals during the 16-week treatment phase (week 4, 8, 12, 16) and again at the 8-week post-treatment follow-up (Table 1). All adverse events will be prospectively recorded, with documentation of their intensity (mild, moderate or severe), duration, outcome, and potential relationship to the study.

### Data management

To ensure impartiality, separate investigators, comprising the data collector, data manager, statistician and outcome assessor, will remain blinded throughout the trial. At week 0 (baseline), participants’ demographic characteristics, symptom severity, medical history, psychological status, and quality of life will be captured via standardized paper questionnaires and electronic surveys. All data will be securely housed on a dedicated network under the supervision of an independent data manager. Two researchers will independently export and assemble the dataset, then perform a cross-verification to confirm completeness and accuracy, any discrepancies will be resolved by a third reviewer referencing the original source documents.

### Data processing and analysis

Descriptive metrics, namely recruitment rates, attrition rates, and treatment adherence, will be reported as count and percentage. All efficacy and safety endpoints will be analyzed under a modified intention-to-treat (ITT) framework. Statistical analyses will be conducted using SPSS for Windows (version 27.0). Statistical significance is defined as a two- sided *P*-value < 0.05. Baseline continuous variables will be expressed as mean ± SD (or median and interquartile range, as appropriate), and categorical variables as frequencies and percentages. Between-group comparisons of normally distributed continuous outcomes will use independent-samples *t*-test, while non-parametric continuous variables will be assessed with Mann-Whitney U test. Categorical data will be evaluated using chi-squared test or Fisher’s exact test. Group differences at weeks 16 and 24 will be tested via analysis of covariance (ANCOVA), adjusting for baseline values. Within-group changes over time will be examined with pair *t*-test for parametric data and Wilcoxon signed-rank test for non-parametric data. Based on the ITT, missing efficacy data will be imputed by last observation carried forward.

### Data and Safety Monitoring Board (DSMB)

Data and Safety Monitoring Board will be established to oversee trial conduct and data integrity. The DSMB will convene at predetermined intervals to evaluate adherence to ethical and safety guidelines, verify the completeness and accuracy of collected data, and review overall study progress. It will also adjudicate adverse event reports and retain the authority to recommend suspension or early termination of the trial if participant safety or data validity is compromised.

### Adverse events

Throughout the trial, the treating acupuncturist will systematically document all AEs. Using a standardized reporting form, each episode’s timing, characteristics, intensity, and presumed cause will be captured. Before each session, the acupuncturist will also actively solicit any delayed reactions since the prior visit. Immediate on-site management will be provided for all events, and participants experiencing serious adverse events will be withdrawn from the study.

## Discussion

This work outlines a single-center, parallel-group trial employing randomization and sham control to rigorously assess the safety and therapeutic efficacy of electroacupuncture for neuropsychiatric manifestations of long COVID. The trial employs strict randomization and blinding procedures to ensure scientific rigor and reliability. The primary and secondary outcomes were chosen to assess multiple dimensions, including cognition, emotion, sleep, and fatigue, allowing for a comprehensive evaluation of the intervention’s potential benefits.

### Scientific basis for acupoint selection

The selection of acupoints plays a crucial role in electroacupuncture treatment. This study selected key acupoints, including GV20 (Baihui), SP6 (Sanyinjiao), and ST36 (Zusanli), based on systematic reviews and expert consensus. Head acupoints are intended to regulate central nervous system functions, while lower limb acupoints emphasize overall balance and meridian flow. The sham control group involves non-acupoint stimulation and mock electrical stimulation to minimize non-specific effects, ensuring comparability between intervention and control groups.

### Multidimensional outcome evaluation

This study combines subjective and objective assessment tools to comprehensively record patient outcomes. Primary outcomes include the MMSE and the CBDI, which reflect changes in cognition and emotion. Secondary outcomes, such as the ISI, BFI-T, and SF-12, further capture changes in sleep quality, fatigue severity, and overall health-related quality of life. The selection and application of these tools ensure sensitivity and accuracy in evaluating outcomes.

### Relevance to clinical practice

Neuropsychiatric symptoms of long COVID significantly affect patients’ quality of life and social participation. However, there is a lack of research on non-pharmacological interventions for these symptoms. This study explores the potential of electroacupuncture as a personalized treatment approach to improve these symptoms. Electroacupuncture is widely used in traditional Chinese medicine. If its efficacy and safety are demonstrated, this study will provide evidence-based support for managing neuropsychiatric symptoms in long COVID. This could enhance clinical acupuncture practices and integrate traditional medicine into modern health management.

### Potential limitations

This trial has certain limitations. First, the treatment schedule and frequency might pose challenges to patient adherence. Second, as a single-center study, the characteristics of participants may reflect only the local population, limiting external generalizability. Additionally, the sham stimulation may not fully eliminate psychological effects, requiring careful consideration during data analysis.

### Strengths and limitations of this study

- This study is a rigorously designed, randomized, patient-assessor-blinded, sham-controlled trial, providing high-level evidence on the efficacy and safety of electroacupuncture for neuropsychiatric symptoms in long COVID patients.
- It employs validated outcome measures ensuring reliable assessment of cognitive, emotional, and sleep-related changes.
- The standardized electroacupuncture protocol, guided by expert consensus and evidence from systematic reviews, ensures consistent intervention across participants and centers.
- The study is limited to long COVID patients in Hong Kong, potentially reducing its generalizability to populations with different healthcare systems or cultural contexts.
- The sham acupuncture design, although validated, may not perfectly mimic the physiological and psychological effects of true acupuncture, potentially introducing bias.
