# Supplementary material for: Electro-acupuncture for long COVID neuropsychiatric symptoms: study protocol for a prospective, randomized sham-controlled, patient-assessor-blinded clinical trial
Source: Front Med (Lausanne). 2025 Sep 4;12:1620288. doi: 10.3389/fmed.2025.1620288 (PMC12443580; doi:10.3389/fmed.2025.1620288)
Supplement: Supplementary file 1 [file Data_Sheet_1.pdf]

Appendix 1

認知功能評估量表

簡易心智量表（Mini-Mental State Examination；MMSE）

於 1975 年 Folstein 及 Mc Huge 所制定，評估項目包括**定向感、注意力、記憶力、語言、口語理解及行為能力、建構力**等項目，評估過程無時間限制，滿分是 30 分，分數越高表示認知功能越好，答對一項給一分，總分若低於24分表示個案有輕度認知功能障礙，若低於 16 分則表示有重度認知功能障礙。目前被廣泛使用。全民健康保險規定診斷為阿茲海默氏症病患藥品給付為 **MMSE** 10~26 分。

**滿分 30 分**	國中以上教育	國小	未受教育
	＜**24** 分 輕度認知功能缺失<br>＜**16** 分 重度認知功能缺失	＜21 分<br>認知功能異常	＜16 分<br>認知功能異常

簡易心智/認知狀態量表(MMSE)

項目	最高分	分數	評分項目
一、定向感 (10)	5<br>5	( )<br>( )	1.時間（5）：幾年？幾月？幾日？星期幾？什麼季節？<br>2 地方（5）：地方：縣/市？醫院？病房？床號？樓層？
二、注意力及計算能力(8)	3	( )	1.訊息登錄（3）：說出三項名詞(例如：房子、汽車、蘋果)：一秒中說一項，說完之後，要求說出這三項名詞，說對一項給一分，請個案記住，等一下會再請他說出這三項名詞。
	5	( )	2.系列減七（5）：由 100 持續減 7，連續減五次答對，一個給一分。(93. 86. 79. 72. 65)<br>如果個案不會計算，則請其執行倒著唸「台南火車站」或「家和萬事興」或 5 個不連續的數字。
三、記憶力 (3)	3	( )	請個案說出剛剛所提的三項名詞。
四、語言(5)	2<br>1	( )<br>( )	1.命名（2）：對筆及錶命名。例：(拿出手錶)這是什麼？<br>2.複誦（1）：請個案覆誦：「白紙真正寫黑字」或「有錢能使鬼推磨」。

	**1**<br>**1**	**(　　)**<br>**(　　)**	**3.理解（1）：給個案看一張上面用大字印著「閉上眼睛」的紙，請個案讀出來，然後照做。**<br>**4.書寫造句（1）：請個案自己寫一句話。**
**五、口語理解及行為能力（3）**	**3**	**(　　)**	**給個案一張空白無圖樣的紙，並且說「用你的右手拿紙（1），對摺（1），然後放在地板上(或再交給我)（1）」。一次說完這三個步驟之後再請個案執行。**
**六、建構力**（1）	**1**	**(　　)**	**圖形抄繪(**請個案將下列交疊的五角形描繪到一張白紙上)<br>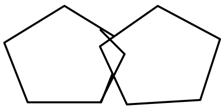

備註：有 11 個題目，30 個答案，達對一題得一分，答錯則不計分，滿分為 30 分，分數位於 24 -30 分為認知功能完整；18-23 分為輕度認知功能障礙，0-17 分為重度認知功能障礙。

Folstein, M., Folstein, S. E., & McHugh, P.(1975).Mini-mental state:a pactical method for grading the cognitive state:a practical method for grading the cognitive state if patient for the clinical.Journal of Psychiatric Research,12(3),189-198.

貝克憂鬱量表

(The Chinese version of the Beck Depression Inventory)

回答方式：請根據個人最近的狀況，選擇一個適當的選項。

1． ☐0 我不感到難過。
☐1 我感覺難過。
☐2 我一直覺得難過且無法振作起來。
☐3 我難過且不快樂，我不能忍受這種情形了。

2． ☐0 對未來我並不感覺特別沮喪。
☐1 對未來我感到沮喪。
☐2 沒有任何事可讓我期盼。
☐3 我覺得未來毫無希望，並且無法改善。

3． ☐0 我不覺得自己是個失敗者。
☐1 我比一般人害怕失敗。
☐2 回想自己的生活，我所看到的都是一大堆失敗。
☐3 我覺得自己是個徹底的失敗者。

4． ☐0 我像過去一樣從一些事中得到滿足。
☐1 我不像過去一樣對一些事感到喜悅。
☐2 我不再從任何事中感到真正的滿足。
☐3 我對任何事都感到煩躁不滿意。

5． ☐0 我沒有罪惡感。
☐1 偶爾我會有罪惡感。
☐2 我常常有罪惡感。
☐3 我總是感到罪惡。

6． ☐0 我不覺得自己正在受罰。
☐1 我覺得自己可能遭受報應。
☐2 我希望受到報應。
☐3 我覺得自己正在自食惡果。

7． ☐0 我對自己並不感到失望。
☐1 我對自己甚感失望。
☐2 我討厭自己。
☐3 我恨自己。

８．☐0 我不覺得自己比別人差勁。
☐1 我對自己的弱點或錯誤常常挑三揀四。
☐2 我總是為了自己的缺失苛責自己。
☐3 祇要出事就會歸咎於自己。

９．☐0 我沒有任何想自殺的念頭。
☐1 我想自殺，但我不會真的那麼做。
☐2 我真想自殺。
☐3 如果有機會，我要自殺。

１０．☐0 和平時比較，我哭的次數並無增加。
☐1 我現在比以前常哭。
☐2 現在我經常哭泣。
☐3 過去我還能，但現在想哭都哭不出來了。

１１．☐0 我對任何事並不會比以前更易動怒。
☐1 我比以前稍微有些脾氣暴躁。
☐2 很多時候我相當苦惱或脾氣暴躁。
☐3 目前我總是容易動怒。

１２．☐0 我關心他人。
☐1 和以前比較我有點不關心別人。
☐2 我關心別人的程度已大不如昔。
☐3 我已不再關心他人。

１３．☐0 我做決定能像以前一樣好。
☐1 我比以前會延後做決定的時間。
☐2 我做決定比以前更感困難。
☐3 我不再能做決定了。

１４．☐0 我不覺得自己比以前差勁。
☐1 我擔心自己變老或不吸引人。
☐2 我覺得自己的外表變得不再吸引人。
☐3 我認為自己長得很醜。

１５．☐0 我的工作情況跟以前一樣好。
☐1 我需要特別努力才能開始工作。
☐2 我必須極力催促自己才能做一些事情。
☐3 我無法做任何事。

１６． □0 我像往常一樣睡得好。
□1 我不像往常一樣睡得好。
□2 我比往常早醒 1 至 2 小時且難再入睡。
□3 我比往常早數小時醒來，且無法再入睡。

１７． □0 我並不比以往感到疲倦。
□1 我比以往易感到疲倦。
□2 幾乎做任何事都令我感到疲倦。
□3 我累得任何事都不想做。

１８． □0 我的食慾不比以前差。
□1 我的食慾不像以前那樣好。
□2 目前我的食慾很差。
□3 我不再感到有任何的食慾。

１９． □0 我的體重並沒有下降，若有，也只有一點。
□1 我的體重下降了 2.5 公斤以上。
□2 我的體重下降了 4.5 公斤以上。
□3 我的體重下降了 7 公斤以上。

２０． □0 我並未比以往更憂慮自己的健康狀況。
□1 我被一些生理病痛困擾，譬如胃痛、便秘等。
□2 我很憂慮自己的健康問題，因此無法顧及許多事務。
□3 我太憂慮自己的健康問題，以致於無法思索任何事情。

２１． □0 最近我對性的興趣並沒有特殊改變。
□1 最近我對性的興趣比以前稍減。
□2 目前我對性的興趣降低很多。
□3 我對性已完全沒有興趣了。

貝克憂鬱量表的計分

當你做完問卷，將二十一題的得分累加起來求出總分。每題最高得分是 3 分，最低是 0 分，因此總分不會高於 63 分，反之，總分最低為 0 分。

貝克憂鬱量表簡易說明

0-13 正常範圍：表示情緒狀態大致平穩。

14-19 輕度憂鬱：表示可能有輕微的情緒波動或低潮，這些低潮尚在其個人可以應付的範圍，但需要他人的關心與支持。

20-28 中度憂鬱：表示有較多的苦惱與煩悶，情緒低潮的處理已經達到個人能夠負荷的範圍，周圍的朋友可以持續地給予關心與支持，然接受專業的協助比較能協助其走出情緒的低潮。

29-63 重度憂鬱：表示情緒低潮可能已經達到憂鬱症的程度，但仍需要專業醫師的診斷再加以確立，再配合藥物治療以利復原；若有心理性因素亦需同時配合諮商治療。這樣的人常常會閃避他人的協助，更需要師長與同儕主動地伸出援手。

Beck AT, Steer RA, Brown GK. Manual for The Beck Depression Inventory Second Edition (BDI-II). San Antonio: Psychological Corporation; 1996a.

失眠嚴重指數
(Insomnia Severity Index)

編號:_______________ 日期: ________________

1. 請評估閣下現時(過去兩星期)失眠問題的**嚴重程度**

	沒有	輕微	普通	嚴重	非常嚴重
入睡困難	0	1	2	3	4
難以維持睡眠	0	1	2	3	4
太早睡醒的問題	0	1	2	3	4

2. 你有幾**滿意**/不滿意你現時的睡眠狀況?

非常滿意				非常不滿意
0	1	2	3	4

3. 你認為你的睡眠問題**妨礙**你日常運作 (例如：日間疲勞、處理工作/日常時務的能力、集中力、記憶、情緒等等) 到那一個程度?

完全沒有妨礙	少許	頗為	非常	極之妨礙
0	1	2	3	4

4. 你的睡眠問題在降低生活質素而言，在其他人眼中有幾**明顯**?

完全不明顯	僅為	頗為	非常	極之明顯
0	1	2	3	4

5. 你對你現時的睡眠問題有幾**憂慮/**苦惱?

完全沒有	少許	頗為	非常	非常大
0	1	2	3	4

Chung KF, Kan KK, **Yeung WF**. Assessing insomnia in adolescents: comparison of Insomnia Severity Index, Athens Insomnia Scale and Sleep Quality Index. *Sleep Med.* 2011;12(5):463-470.

台灣版簡明疲憊量表
(Brief Fatigue Inventory-Taiwan)

在我們生活中，大多數我們有時會感到疲倦或疲憊。在過去一週中，您是否曾感到不尋常的疲倦或疲憊？是　　　　　否

1.請在下面數字中圈選一個，以表示現在的疲憊(疲倦、疲勞)程度

無疲憊　0　1　2　3　4　5　6　7　8　9　10　能想像最嚴重的程度

2.請在下面數字中圈選一個，以表示過去 24 小時中一般疲憊(疲倦、疲勞)的程度

無疲憊　0　1　2　3　4　5　6　7　8　9　10　能想像最嚴重的程度

3.請在下面數字中圈選一個，以表示過去 24 小時中疲憊(疲倦、疲勞)最劇烈時的程度

無疲憊　0　1　2　3　4　5　6　7　8　9　10　能想像最嚴重的程度

4.請在下面數字中圈選一個，以表示過去 24 小時中，疲憊(疲倦、疲勞)影響您的程度

(1)一般活動

無疲憊　0　1　2　3　4　5　6　7　8　9　10　能想像最嚴重的程度

(2)情緒

無疲憊　0　1　2　3　4　5　6　7　8　9　10　能想像最嚴重的程度

(3)行走能力

無疲憊　0　1　2　3　4　5　6　7　8　9　10　能想像最嚴重的程度

(4)日常工作(包括外出工作及家事)

無疲憊　0　1　2　3　4　5　6　7　8　9　10　能想像最嚴重的程度

(5)與人交往

無疲憊　0　1　2　3　4　5　6　7　8　9　10　能想像最嚴重的程度

(6)生活樂趣

無疲憊　0　1　2　3　4　5　6　7　8　9　10　能想像最嚴重的程度

Wang XS, Hao XS, Wang Y, et al. Validation study of the Chinese version of the Brief Fatigue Inventory (BFI-C). J Pain Symptom Manage. 2004;27(4):322-332. doi:10.1016/j.jpainsymman.2003.09.008

生活質量調查簡表 12 條 (the Short Form 12)

1.總體來說，您認為您現在的健康狀況是？[單選題]
○非常好(100 分)
○很好(75 分)
○好(50 分)
○一般（不好不差）(25 分)
○差(0 分)

2.中等強度的活動，例如搬動桌子，用吸塵機吸塵或者清潔地板，打保齡球，或者打太極拳？[單選題]
○有很大限制(0 分)
○有一點限制(50 分)
○沒有任何限制(100 分)

3.上三層樓梯？[單選題]
○有很大限制(0 分)
○有一點限制(50 分)
○沒有任何限制(100 分)

4.在過去四個星期裡，您會否因為身體健康的原因而令您在工作或日常活動中實際完成的比想做的少？[單選題]
○會(0 分)
○不會(100 分)

5.在過去四個星期裡的工作或日常活動中，您會否因為身體健康的原因而令您的工作或活動受限制？（種類）[單選題]
○會(0 分)
○不會(100 分)

6.在過去四個星期裏，您會否因為情緒方面的原因（比如感到沮喪或焦慮）而令您在工作或者日常活動中實際完成的比想做的少？[單選題]
○會(0 分)
○不會(100 分)

7.在過去四個星期裡的工作或日常活動中，您會否因為情緒方面的原因（比如感到沮喪或焦慮）而令您的工作或活動受限制？（種類）[單選題]
○會(0 分)
○不會(100 分)

8.在過去四個星期裡，您身體上的疼痛對您的日常工作（包括上班和家務）有多大影響？[單選題]

○完全沒有影響(100 分)
○有很少影響(80 分)
○有一些影響(60 分)
○有較大影響(40 分)
○有非常大的影響(20 分)
○不適用(0 分)

9.在過去四個星期裏，您有多少時間感到心平氣和？[單選題]
○常常(100 分)
○大部分時間(80 分)
○很多時間(60 分)
○一般(40 分)
○只有很少時間(20 分)
○從來沒有(0 分)

10.在過去四個星期裏，您有多少時間感到精力充足？[單選題]
○常常(100 分)
○大部分時間(80 分)
○很多時間(60 分)
○一般(40 分)
○偶爾有(20 分)
○從來沒有(0 分)

11.在過去四個星期裏，您有多少時間感到心情不好，悶悶不樂或沮喪？[單選題]
○常常(0 分)
○大部分時間(20 分)
○很多時間(40 分)
○一般(60 分)
○偶爾有(80 分)
○從來沒有(100 分)

12.在過去四個星期裏，有多少時間由於您身體健康或情緒問題而妨礙了您的社交活動（如探親或訪友）[單選題]
○常常(0 分)
○大部分時間(20 分)
○很多時間(40 分)
○一般(60 分)
○偶爾有(80 分)
○從來沒有(100 分)

Ware J Jr, Kosinski M, Keller SD. A 12-Item Short-Form Health Survey: construction of scales and preliminary tests of reliability and validity. Med Care. 1996;34(3):220-233. doi:10.1097/00005650-199603000-00003
