# Supplementary material for: Electro-acupuncture for long COVID neuropsychiatric symptoms: study protocol for a prospective, randomized sham-controlled, patient-assessor-blinded clinical trial
Source: Front Med (Lausanne). 2025 Sep 4;12:1620288. doi: 10.3389/fmed.2025.1620288 (PMC12443580; doi:10.3389/fmed.2025.1620288)
Supplement: Supplementary file 2 [file Data_Sheet_2.pdf]

SCHOOL OF CHINESE MEDICINE HONG KONG BAPTIST UNIVERSITY
INFORMED CONSENT STATEMENT

Title: Electro-acupuncture for long covid neuropsychiatric symptoms: a prospective, randomized sham-controlled, double-blinded clinical trial

Study Background

As of 16 February 2022, the global coronavirus disease 2019 (COVID-19) pandemic has caused more than 417 million infections and 5.8 million deaths. Neurological complications have emerged as a significant cause of morbidity and mortality in the ongoing COVID-19 pandemic. Beside respiratory insufficiency, many hospitalized patients exhibit neurological manifestations ranging from headache and loss of smell, to confusion and disabling strokes. Furthermore, maladies such as impaired concentration, headache, sensory disturbances, depression, and even psychosis may persist for months after infection, as part of a constellation of symptoms now called Long Covid. Even young people with mild initial disease can develop acute COVID-19 and Long Covid neuropsychiatric syndromes.

Acupuncture is a common technique for treating neuropathy in traditional Chinese medicine. Manual and electrical stimulation on the acupoints are commonly used stimulation modes of acupuncture. Increasing evidence shows that electroacupuncture could reduce the functional deficits of neurophathy and prevent cognitive deficiency involved in brain diseases. Pei and colleagues showed that electroacupuncture could significantly improve spatial memory impairment.

During the COVID-19 epidemic, acupuncture has been used as a complementary treatment for it in China. An observational clinical study found the effectiveness of acupuncture combined with routine regimens in treating COVID-19, all 33 COVID-19 patients (including five patients with severe illness) were cured and discharged, with significantly relieved symptoms of fatigue, anxiety, and insomnia.
Notably, the guideline for acupuncture and moxibustion intervention on COVID-19 (Second edition) has been recently proposed and issued by Chinese association of acupuncture and moxibustion.

Although a number of randomized controlled trials (RCTs) and systematic reviews and meta-analyses are currently being concerning efficacy and safety of acupuncture in treating COVID-19 are under way, there is not RCT about investigating the efficacy and safety of electro-acupuncture compared to sham acupuncture for treatment of long covid neuropsychiatric symptomse. Therefore, evidence of the

efficacy and safety of electroacupuncture for the treatment of neuropsychiatric symptoms of prolonged coronavirus disease is lacking, whereas acupuncture is likely to be an effective and safe way to improve such symptoms. High-quality randomized controlled trials are necessary to find effective treatments for neurological symptoms in patients discharged from hospitals with COVID-19.

Study Aims

We plan to conduct this study to investigate the efficacy and safety of electro-acupuncture compared to sham acupuncture for treatment of long covid neuropsychiatric symptoms.

Study Plan

This is a prospective, randomized, sham-controlled, double-blinded and multiple center trial; 150 participants are estimated to be eligible for the Program. The participants will be assessed for cognitive functions, depressive/ anxiety syndrome, and quality of sleeping and life.

The participants will receive four months acupuncture treatment (2 session per week) under the Rehabilitation Program organized by CMCTRs, you are eligible for the study. After the four months treatment, you will enter the follow-up period. Each participant will be assessed every month during the treatment and two months after treatment as follow-up.

Potential Benefits

This is a clinical trial, Your participation must have contributed to evaluate the efficacy and safety of electro-acupuncture for treatment of long covid neuropsychiatric symptoms, with the aim to share the experience in clinical practice and facilitate scientific development of acupuncture treatment in the fierce battle of COVID-19.

Potential Risks

Since the participants are under the Rehabilitation Program in CMCTRs, adverse events after taking the acupincture treatment will be managed by CMCTRs. In this study, there will have a very low risk of doing the questionnaires. Moreover, if you feel unwell after acupuncture, including pain and bleeding from the needle insertion, and dizziness, the treatment will be stopped immediately, and further medical treatment or referral to Mr. and Mrs. Chan Hon Yin Modern Chinese Medicine Research and Service Centre will be arranged, if necessary. The study is also covered

by professional liability insurance policy.

Responsibilities

Your participation in this research is voluntary. Whether to participate in the study depends on your decision. Even if you refuse to participate, you will still receive the medical services and care you deserve. You can terminate your participation during the study period and withdraw without giving a reason.

If you agree to participate in this study, you need to provide your personal information, including name, age, address, telephone number and medical history related to your COVID-19 infection. Moreover, within the study period, you need to be assessed by our investigators every month according to the research schedule.

Confidentiality

Your personal data will be used research purposes only, which including Hospital Authority and the other two universities who are involved in carrying out the study together with the Hong Kong Baptist University. All the data will be destroyed after 7 years of the completion of the research study.

Compensation and Costs

There is no compensation or additional costs to the participant that may result from participation in the research. Any test and/or consultation fee is free. After the completion of this study, you can still seek for medical treatment in our clinics at your own cost.

Contact

If you have questions at any time about the study or the procedures, you may contact our researcher.

Principle Investigator: Linda Zhong
Phone: 34116523
Fax: 34112929
E-mail: ldzhong@hkbu.edu.hk

If you feel that your rights as a participant in the study have been violated during the course of the study, you may contact the representative of HKBU Research Ethics Committee by email at : hkbu_rec@hkbu.edu.hk.

Participation

Your participation in this study is voluntary. If you decide to participate, you may withdraw from the study at any time without penalty and without loss of benefits to

which you are otherwise entitled. If you withdraw from the study before data collection is completed your data will be destroyed.

Consent

I have read and understand the above information. I have received a copy of this form. I agree to participate in this study.

Subject's signature_________________ Date _______________________

Investigator's signature______________ Date ______________________
