# Supplementary material for: Electro-acupuncture for long COVID neuropsychiatric symptoms: study protocol for a prospective, randomized sham-controlled, patient-assessor-blinded clinical trial
Source: Front Med (Lausanne). 2025 Sep 4;12:1620288. doi: 10.3389/fmed.2025.1620288 (PMC12443580; doi:10.3389/fmed.2025.1620288)
Supplement: Supplementary file 3 [file Data_Sheet_3.pdf]

# Acupoint of treatment group

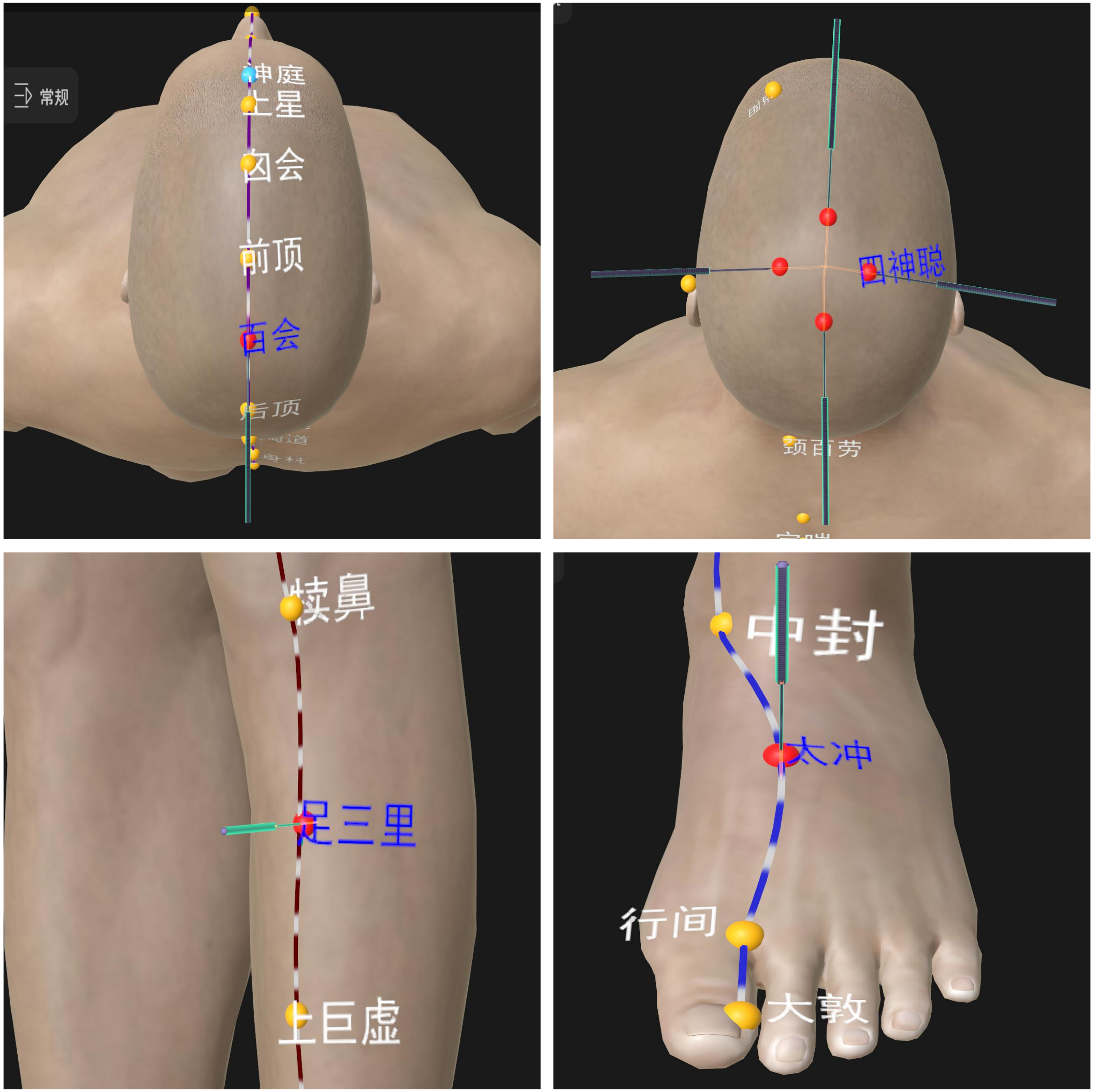


ps: red point represented the acupoint of treatment group

# Non-acupoint with corresponding of sham acupuncture group

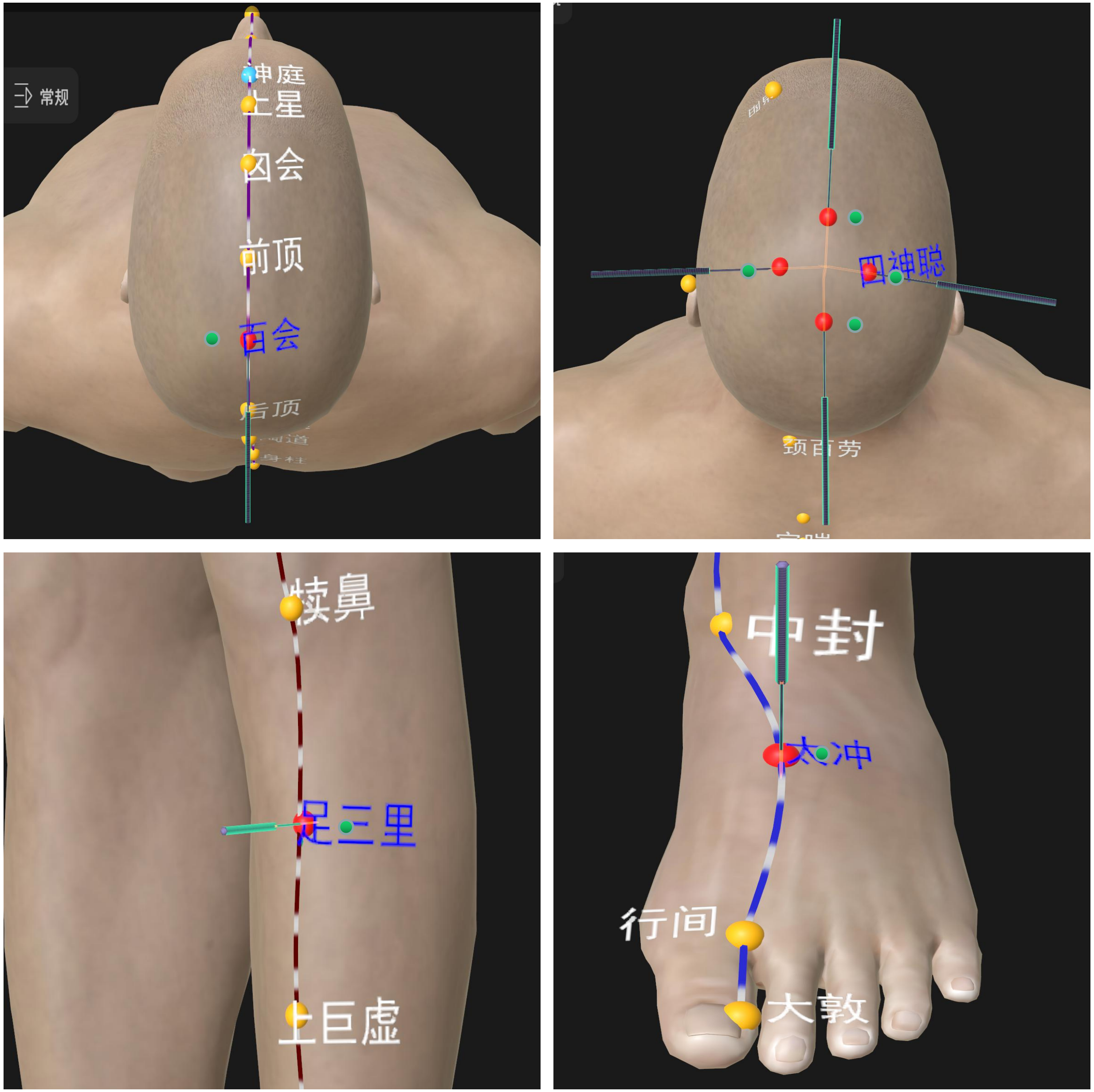


ps: green point represented the non-acupoint with corresponding of sham acupuncture group
